# Dietary supplementation of Nile tilapia (*Oreochromis niloticus*) with β-glucan and/or *Bacillus coagulans*: Synergistic impacts on performance, immune responses, redox status and expression of some related genes

**DOI:** 10.3389/fvets.2022.1011715

**Published:** 2022-09-23

**Authors:** Ahmed F. Fath El-Bab, Kamlah A. Majrashi, Huda M. Sheikh, Manal E. Shafi, Ibrahim T. El-Ratel, Ahmed N. F. Neamat-Allah, Ali A. El-Raghi, Amar Y. Abd Elazem, Mohamed F. Abd-Elghany, Sameh A. Abdelnour, Maisa S. Abduh, Mariusz Jaremko, Mohammed A. E. Naiel

**Affiliations:** ^1^Department of Animal Production, Faculty of Agriculture, Damietta University, Damietta, Egypt; ^2^Biological Sciences Department, College of Science & Arts, King Abdulaziz University, Rabigh, Saudi Arabia; ^3^Department of Biology, College of Science, University of Jeddah, Jeddah, Saudi Arabia; ^4^Department of Biological Sciences, Zoology, King Abdulaziz University, Jeddah, Saudi Arabia; ^5^Department of Poultry Production, Faculty of Agriculture, Damietta University, Damietta, Egypt; ^6^Department of Clinical Pathology, Faculty of Veterinary Medicine, Zagazig University, Zagazig, Egypt; ^7^Department of Animal Production, Faculty of Agriculture, Al-Azhar University, Cairo, Egypt; ^8^Department of Animal Production, Faculty of Agriculture, Zagazig University, Zagazig, Egypt; ^9^Department of Medical Laboratory Sciences, Faculty of Applied Medical Sciences, King Abdulaziz University, Jeddah, Saudi Arabia; ^10^Center of Excellence in Genomic Medicine Research, King Abdulaziz University, Jeddah, Saudi Arabia; ^11^Smart-Health Initiative and Red Sea Research Center, Division of Biological and Environmental Sciences and Engineering, King Abdullah University of Science and Technology, Thuwal, Saudi Arabia

**Keywords:** *Bacillus coagulans*, growth, tilapia, immunity, prebiotic

## Abstract

A 14-week feeding study was conducted to assess the effects of feed supplementation with prebiotics β-glucan (BG group) and/or probiotics *Bacillus coagulans* (BC group) on *O. niloticus* growth performance, body analysis, intestinal structure, immunological response, and antioxidant status. The fish were equally divided into six groups, as follows: the fish group fed an un-supplemented diet served as a control group; the other fish groups were fed supplemented diets with 0.1 g β-glucan kg^−1^; 1 g *Bacillus coagulans* kg^−1^; 2 g *B. coagulans* kg^−1^; 0.1 g β-glucan combined with 1 g *B. coagulans* kg^−1^; 0.1 g β-glucan combined with 2 g *B. coagulans* kg^−1^. The findings revealed that supplementing *B. coagulans* and the β-glucan mixture improved growth performance and feed efficiency parameters (RGR and SGR) more than the other groups. The fish flesh analysis revealed increased crude protein and dry matter content and lower lipid and ash levels in the BG and BC supplemented groups than in the Control group. On the other hand, β-glucan and *B. coagulans* supplementation significantly boosted antioxidant activity and immunological responses in serum as determined by CAT, MDA, lysozyme, and phagocytic activity. Dietary β-glucan and *B. coagulans* supplementation remarkedly enhanced anterior intestine villus histomorphometry characteristics. Furthermore, *B. coagulans*, alone or in combination with β-glucan, could reduce *HSP70* and *IL-1*β gene expression while increasing *IL-8* and *GH* gene expression. According to the findings, *B. coagulans* and/or BG increased growth performance by increasing gut health and morphology. Furthermore, β-glucan and *B. coagulans* supplementation enhanced Tilapia's body composition, immunological responses, and antioxidant status.

## Introduction

Over the previous several decades, a broad range of chemicals like antiseptics, antibiotics, and antiparasitic agents have been applied in the production of sustainable aquaculture industry (1). Worldwide, antibiotics of various types are widely used for treating and controlling bacterial infections. However, this raises the risk of resistant pathogenic bacteria, antibiotic accumulation in fish flesh, and food safety risks for human health (2, 3). Nowadays, the theory of free-antibiotic aquaculture has been generally approved in the aquatic industry and worldwide. Furthermore, alternative antibiotic chemicals like prebiotics and probiotics have been widely accepted because they can improve general health status, alleviate stress threats and reduce infectious diseases in several aquatic fish species (4–6). Although, the prebiotic and probiotic enriched fish feed has been extensively investigated in various fish species, little is known about the influence of synergistic combinations of pro- and prebiotics on the productivity and immunity of farmed fish.

Amongst the most widespread prebiotics are β-glucans, polysaccharides found in the cell walls structure of bacteria, fungi, and plants (7). The β-glucans applied in aquatic feeds are derived mostly from *Saccharomyces cerevisiae* and have a linear structure of β-1,3 connected D-glucopyranosyl units with β-1,6 bending glucose side-chains (8). Recently, β-glucan has been extensively used as an effective immunostimulant due to its ability to stimulate the immune system by boosting the binding activity of specific receptors with macro and micro-phages, neutrophils, and natural killer (NK) cells. However, the exact mode of action is still unknown (9). In addition, β-glucan could have several other biological activities such as antibacterial, antioxidant and antitoxic (10). It is well known that β-glucan supplementation might improve immunological responses of several species of aquatic creatures such as *Oncorhynchus mykiss* (11), *Trachinotus ovatus* (12), *Cyprinus carpio* (13), *Lutjanus peru* (14) and *Litopenaeus vannamei* (15). Likewise, previous reports investigated that the inclusion β-glucan within fish feed could stimulate the performance of some fish species such as *Cyprinus carpio* (16), *Pseudosciaena crocea* (17), *Oncorhynchus mykiss* (18) and *Apostichopus japonicas* (19). In contrast, some studies have found no significant influence of β-glucan dietary administration on the performance of *Oreochromis niloticus* (20, 21) and *Dicentrarchus labrax* (22). While, Dawood et al., (23) demonstrated that β-glucan dietary administration stimulated the lysozyme and phagocytic activities in Nile tilapia However, increasing the efficacy of employing β-glucan by combining various probiotic strains has not been explored.

On the other hand, probiotic strains, even live or dead bacteria might promote intestinal microbiota homeostasis by influencing the interspecific interaction within the entire microbial population (24). According to the data from previous research, dietary probiotic administration might promote general host health status *via* influencing organ development, nutrition metabolism, and the immune response (4, 25, 26). Many substances in the bacterial cell walls may stimulate the host's immune system and boost innate and adaptive immunity against infectious pathogens (27). Nowadays, many probiotics products are applied in aquaculture that contain bacterial strains such as *Lactobacillus* sp., *Bifidobacterium* sp., *Bacillus* spp., *Pseudomonas* sp., and others (5, 28, 29). Among *Bacillus* spp., *Bacillus coagulans* is the most extensively employed of these strains (30). It has a beneficial influence on the immunological activity and growth of several aquatic animals such as common carp (31), grass carp (32), and white shrimp (33). Furthermore, several research have shown that probiotic dietary supplementation can stimulate growth and immune response in *Oreochromis niloticus* (34, 35). Specifically, previous reports have implied that dietary supplementation with *B. coagulans* can promote growth, immune response and disease resistance in white shrimp (36) and common carp (37).

Tilapia, a cichlid fish species, is a main economic freshwater fish extensively cultivated in Egypt. Recently, disease outbreaks have become increasingly prevalent in farmed fish due to the expansion of intensive aquaculture and worsening of water physiochemical features. Many studies have investigated the biological influences of β-glucan or *B. coagulans* on some fish species, but little is known about their combined effects in Tilapia. Therefore, the main objective of the current trial was to evaluate the synergistic benefits of β-glucan combined with *B. coagulans* inclusion in tilapia diets that enhanced β-glucan utilization, which affects growth performance intestinal histomorphometry, immune activity, antioxidant status and regulation of some related gene expression of Tilapia.

## Materials and methods

### Tested additives

In this experiment, two available commercial products were employed. *B. coagulans* DSM 32016 (Technospore^®^ Biochem co., Germany) commercial products containing 2.5 × 10^9^ CFU/g have been used as a safe feed additive. Also, β-glucan powder extract (Batch No: 2809115, Pharma Health Co. Egypt) was applied as a prebiotic compound.

### Experimental design and rearing conditions

Three hundred healthy *O. niloticus* fingerlings (6.95 ± 0.15 g) were purchased from a private hatchery in Fowa, Kafrelsheikh governate, Egypt, and transported into the wet Laboratory of the Faculty of Agriculture, Damietta University, Egypt, using plastic bags containing sufficient purified oxygen. For 2 weeks, the transported fish were kept in a 2,000 L fiberglass tank to acclimate to Laboratory conditions. The fish were then randomly assigned to six equal groups within each of the five replicates in a glass aquarium with ten fish. Fish were fed to apparent satiation three times daily (8:00, 13:00, and 16: 00) for 14-weeks. Throughout the feeding trial period, the fish rearing conditions and the water physiochemical features (mean ± SD) were preserved at the optimum conditions for tilapia; aquarium dimensions: 40 × 60 × 70 cm, photoperiod 12 h L:12 h D, water temperature (26.5 ± 1°C), pH (7.2 ± 0.5), dissolved oxygen (7.3 ± 0.5 mg/L) and total ammonia as nitrogen (<0.3 mg/L). The tested level of β-glucan in this experiment was a 0.1 g β-glucan kg^−1^ diet as endorsed by the manufacturer's instruction and previous reports (20, 38). The *B. coagulans* product levels examined in the present trial were decided to be within the sufficient average (1 or 2 g kg^−1^ diet) based on the Xu et al. (31) report. The feeding study was extended to 14 weeks. While the fish were divided into six equal groups, as follows:

CNT, the fish group fed un-supplemented diet (Control group);

BG, the fish group fed basal diet supplemented with 0.1 g β-glucan kg^−1^;

BC1, the fish group fed basal diet supplemented with 1 g *B. coagulans* kg^−1^;

BC2, the fish group fed basal diet supplemented with 2 g *B. coagulans* kg^−1^;

BG+BC1, the fish group fed basal diet supplemented by 0.1 g β-glucan combined with 1 g *B. coagulans* kg^−1^;

BG+BC2, fish group fed basal diet supplemented with 0.1 g β-glucan combined with 2 g *B. coagulans* kg^−1^.

### Feed ingredients and diet preparation

The formulated diets ingredients, which are commonly applied in tilapia rations, were Fishmeal (CP, 62%), Soybean meal (CP, 44%), Wheat bran (CP, 16%), Yellow corn (CP, 4%) and Corn gluten meal (CP, 60%). The basal ration (Table 1) was prepared to meet the essential nutrient requirements of tilapia fish as ascribed by NRC (39) guidelines. Each prepared diet was produced by thoroughly combining all of the ingredients. After that, 200 ml of water per kilogram diet were blended, and the obtained combination (ingredients, feed additive, and water) was homogenized to produce a suitable mixture for each diet. Each diet was pelleted *via* a laboratory pellet machine with a 1 mm diameter die. Then, the produced wet pellets were left to completely dry at room temperature. The dried pellets were kept in dark plastic bags and then preserved in the refrigerator at −4°C until use. The viable count and activity of the probiotic bacteria in the prepared diets were examined after 24 h of storage following Kumaree et al. (40) procedure. Proximate analysis of the examined diets was shown in Table 1, and the chemical composition of formulated diet samples was estimated owing to AOAC (41) procedures.

**Table 1:** Formulation and chemical composition of the basal diet (% dry matter).

**Items**	**CNT**	**BG**	**BC1**	**BC2**	**BG+BC1**	**BG+BC2**
Fishmeal (62%)	8	8	8	8	8	8
Soybean meal (44%)	37	37	37	37	37	37
Wheat bran (16%)	12	12	12	12	12	12
Yellow corn (4%)	26	26.9	25	24	24.9	23.9
Corn gluten meal (60%)	10	10	10	10	10	10
Fish oil	4	4	4	4	4	4
Mineral^a^ and Vitamin^b^ premix	3	3	3	3	3	3
*Bacillus coagulans* g/kg	0	0	1	2	1	2
*β-*glucan	0	0.1	0	0	0.1	0.1
Total	100	100	100	100	100	100
Crude protein	30.2	30.16	30.12	30.22	30.18	30.16
Dry matter	90.1	89.8	89.5	89.7	89.1	89.2
Crude lipid	6.3	6.4	6.5	6.3	6.4	6.2
Fiber	5.1	5.2	5.1	5.2	5	5.4
Ash	6.3	6.1	6.4	6.2	6.5	5.1
NFE^c^	46.78	46.46	46.9	46.54	46.82	45.81
Gross energy, MJ/kg	442.571	443.023	442.951	442.201	442.4478	445.7824

^a^Composition of mineral premix kg^−1^: manganese,53 g; zinc,40 g; iron, 20 g; copper, 2.7 g; iodine, 0.34 g; selenium, 70 mg; cobalt, 70 mg and calcium carbonate as carrier up to 1 kg.

^b^Composition of vitamin premix kg^−1^: vitamin A,8,000,000 IU; vitamin D_3_, 2,000,000 IU; vitamin E, 7,000 mg; vitamin K_3_,1,500 mg; vitamin B_1_, 700 mg; vitamin B_2_, 3,500 mg; vitamin B_6_, 1,000 mg; vitamin B_12_, 7 mg; biotin, 50 mg; folic acid, 700 mg; nicotinic, 20,000 mg; pantothenic acid,7,000 mg.

^c^NFE = 1000 – (Crude Protein + Crude Lipids+ ash + Crude Fiber).

### Growth parameters and efficiency of feed

Survived fish were fed and weighed biweekly to estimate actual consumed diets and growth. For assessing the performance and efficiency of the consumed diet of fish, different parameters such as Cumulative body weight gain (CBWG), Average daily gain (ADG), Specific growth rate (SGR), Relative Growth Rate (RGR), Feed Conversion Rate (FCR) and Protein Efficiency Ratio (PER) were taken into consideration and were determined applying the following formula.

Average daily gain (ADG) = $\frac{W2-W1}{T}$,

Specific growth rate (SGR) $=\frac{\left(L_{n}w_{2}-L_{n}w_{1}\right)}{T}\times \;100$,

Relative growth rate (RGR)= $\frac{\left(W2-W1\right)}{W2}\times \;100$.

Feed conversion ratio (FCR) $=\frac{TFI}{W2-W1}$,

Protein efficiency ratio (PER)= $\frac{W2-W1}{PI}$,

Where W_1_ and W_2_ are the initial and final biomass, respectively. TFI, PI, and T are the total consumed feed, total protein intake and the total number of feeding trial days (14-weeks), respectively.

### Fish body chemical analysis

Six fish from each treatment were transferred into plastic bags and stored at −20°C to determine the proximate analysis of the total fish body. The crude protein, lipid, and ash content of the fish body were determined using the Association of Official Analytical Chemists standard techniques (41). The moisture content was determined by drying the samples until obtaining a consistent weight using a drying oven (GCA, model 18 EM, Precision Scientific group, Chicago, IL, USA) at 85°C for 24 h.

### Blood sampling protocol

Fish were anesthetized with 100 μg ml^−1^ MS222 (Tricaine methane-sulfonate, Sigma- Aldrich Co. LLC.) before blood collection. Blood samples were collected from two fish from each aquarium (ten fish per treatment) at random (42). Blood samples were taken from the caudal vein using 2.5 ml sterile syringes and split into equal parts. The first part was stored in a heparinized tube for hematological measurements. In contrast, the second portion was allowed to clot for 30 mins at ambient temperature before being stored in a refrigerator at 4°C for 3 h. Afterward, the clotted samples were centrifuged at 3,000 rpm for 10 mins at 4°C to extract serum, which was kept at −20°C until further biochemical, antioxidant, and immunological investigation. All samples have always been analyzed within 30 days after being kept frozen.

### Blood hematological assessments

Following Stoskopf (43) procedure, the erythrocytes and leukocytes counts were estimated using a hemocytometer and Natt-Herrik solution. While, the hemoglobin level was determined using the cyanmethaemoglobin procedure as endorsed by Balasubramaniam and Malathi (44). Moreover, the microhematocrit method was applied for estimation of the PCV% and calculation MCV, MCH, and MCHC (45). To determine the differential leukocytic count, blood smear slides were prepared, air dried, fixed with methanol for 3–5 min, stained with Giemsa stain for 8–10 min, washed with distilled water, and after left to dry under room temperature following Blaxhall and Daisley (46) technique.

### Blood biochemical analysis

Blood protein content (TP: total protein; ALB: albumin) was estimated *via* colorimetrical technique, whereas globulin (GB) level was determined by subtracting albumin value from total protein concentration. Moreover, liver function enzymes (ALT: alanine transaminase; AST: aspartate transaminase) were estimated using commercial kits (Assay Kit, 384 well, Colorimetric/Fluorometric, ABACM241035) following Wilkinson et al., (47) procedure. In addition, triglycerides, cholesterol and glucose concentrations were measured using commercial kits (Bio-Diagnostic Co. Egypt) by applying colorimetric methods following Fossati and Prencipe (48), Richmond (49) and Caraway (50) procedures, respectively.

### Serum antioxidant and immunity parameters

The activity of serum oxidative remarks, including catalase (CAT), superoxide dis-mutase (SOD) and malonaldehyde (MDA) was estimated using the specific-commercial kits (Bio-diagnostic Co., Egypt). While, Cortisol level was assessed using Fluorescence Immunoassay rapid quantitative procedure applying a specific-commercial kit and FIA meter (Finecare, FIA meter plus, Guangzhou, Wondfo, Biotech. Co., China). The determination process was operated owing to product guidelines.

Immunoglobulin M (IgM) level was estimated via applying ELISA technique using a commercial kit (Fish Immunoglobulin M, ELISA Kit, Cat.No:MBS042385, My-BioSource, Co., Southern California, San Diego, USA) as ascribed by Wuthiekanun et al. (51). The lysozyme activity was evaluated using the *Micrococcus lysodeikticus* (Sigma, USA) technique at 450 nm using microplate ELISA reader as described by Demers and Bayne (52) procedure. The Cai et al. (53) method was applied to assess leukocyte phagocytic function activity. Furthermore, the used bacterial strain (*Aeromonas hydrophila*, 1 × 10^6^) was obtained from the Department of Fish Diseases and Management, Sakha Aquaculture Research Unit, Central Lab. For Aquaculture Research, A.R.C., Egypt. The applied bacterial strain was identified using morphological and biochemical features, as described by Joseph and Carnahan (54).

### Intestinal histological description, histomorphometric, and digestive enzymes analysis

The intestinal samples were eviscerated, weighed to 1 g, and blended with an appropriate quantity of phosphate buffer saline (PBS) solution (at a weight ratio of 1 g sample per 4 ml PBS) according to Kiernan (55) method and then transferred into a 10 mL Eppendorf tube. After that emulsified completely with an electric homogenizer in ice water bath for 15 s. Samples were centrifuged at 12,000 rpm at 4°C for 20 min. Lastly, the liquid supernatant was isolated for digestive enzymes estimation. The activity of the digestive enzymes, including Lipase (REF:281 001 Spectrum, Egyptian Co. Biotechnology, Egypt) and Amylase (CAT. NO. AY 10 50 Bio-diagnostic Co. Egypt) were estimated using commercial kits. The analysis procedure was directed according to the manufacture's instruction.

Anterior intestines of three fish per aquarium were gathered, removed, retained in Bouin's fixative solution for 24 h and then reserved in 70% absolute ethanol. Prepared samples for light microscopy were dehydrated in a graded concentration of ethanol, then cleared in xylene and fixed in paraffin wax. Hereafter, approximately 0.5 cm length segments of the anterior intestine were sliced transversely into 5 μm sections by microtome (Manually operated Rotary Microtome CUT 4055, D-69190 Walldorf, Germany) and stained with haematoxylin and eosin (HE) pigments. The prepared slides were inspected under a light microscope (Nikon, Tokyo, Japan) to determine the morphological parameters. The length, width of intestinal villi and crypt depth, inter-villi space and goblet cell count were estimated using image analysis software (NIH, Bethesda, MD) (30 measurements per fish, three fish per aquarium). The villi surface area was computed following Sakamoto et al. (56) formula.

### RNA extraction, and quantitative real-time PCR

Total RNA was isolated and extracted from the liver samples using Trizol reagents (iNtRON Biotechnology) according to the manufacturer's instructions. To prevent RNA contamination, 2 μl of RNase was blended with 20 μl of DNA dissolved in Tris-buffer solution (pH = 8.0) and incubated for 3–4 h at 37°C. Then, RNA concentration was determined by Nanodrop (Quawell, USA). Real-time PCRs were achieved for selected genes, including interleukin-8 (*IL-6*), interleukin-*1*β (*IL-1*β), heat shock protein (*HSP70*), and growth hormone (*GH*). The β-actin and *GAPDH* genes were the main housekeeping genes to normalize cDNA loading. The primers employed in the present study are illustrated in Table 2. Real-time PCR amplifications were performed following the Pereira-Gomez et al. (57) procedure using SensiFast SYBR Lo-Rox kit (Bioline) in 20 μl reaction mixtures containing 2 μl of cDNA, the gene-specific primers (0.5 μM each); and SYBR 10 μl. The conditions for the thermal cycling were initial denaturation at 95°C for 10 min, followed by 40 cycles at 95°C for 15 s and 60°C for 1 min. The estimated genes were performed in triplicate. The fold change was estimated using the 2^−Δ*ΔCT*^ formula (58).

**Table 2:** The sequences of applicable primers used for real-time q-PCR investigation of gene expression.

**Gene**	**Forward 5^′^->3^′^**	**Reverse 5^′^->3^′^**	**Accession no**.
*HSP70*	TGGAGTCCTACGCCTTCAACA	CAGGTAGCACCAGTGGGCAT	KP645179
*GH*	CTGGTTGAGTCCTGGGAGTT	CAGGTGGTTAGTCGCATTGG	KT387598.1
*IL-8*	GCACTGCCGCTGCATTAAG	GCAGTGGGAGTTGGGAAGAA	XM_003447521
*IL-1β*	AGAGCAGCAATTCA GAGC	GTGCTGATGTACCAGT	XM_005457887.3
*GAPDH*	CCGATGTGTCAGTGGTGGAT	GCCTTCTTGACGGCTTCCTT	NM_001279552.1
β-Actin	CAGCAAGCAGGAGTACGATGAG	TGTGTGGTGTGTGGTTGTTTTG	XM_003443127

### Ethical approval statements

This *in-vivo* study was carried out in faithful agreement with the ethical guidelines of the Ethical principles of the Experimental Animal Welfare Ethics Committee of Zagazig University. The protocol was approved by the Committee on Research Ethics of the department of Animal Production, Zagazig University. Whereas, all efforts were applied to minimize the suffering and painful for experimental fish.

### Statistical procedure

The normality and homogeneity of data were examined using Shapiro-Wilk test. Furthermore, all computed and estimated data were subjected to a one-way ANOVA statistical analysis method and differences between the means were verified by Tukey range test. The tested level of significance was set at *P* < 0.05. The results are presented as mean ± pooled SE values and all statistical examinations were achieved using SPSS 22.0 (SPSS Inc., 2013, USA).

## Results

### Growth performance and feed utilization

At the end of a 14-week feeding trial, compared to the control group administered un-supplemented diets, the fish groups fed BG plus BC2 had markedly higher final biomass, cumulative body gain, and average daily growth (*P* > 0.001), as well as a higher protein efficiency ratio (Table 3). Furthermore, dietary supplementation with β-glucan in combination with any dose of *B. coagulans* could significantly improve SGR and RGR compared to the control group (*P* < 0.001). Even though there was no significant variation in FCR values due to the effects of feed additives, the lowest FCR values were recorded in the BC2 groups alone or in combination with BG.

**Table 3:** Effects of dietary supplementation with β-glucan and/or *Bacillus coagulans* on performance, and efficiency of feed of tilapia fish.

**Parameters**	**Dietary treatments**						**Pooled *SEM***	***p-*value**
	**CNT**	**BG**	**BC1**	**BC2**	**BG+BC1**	**BG+BC2**		
IBW (g)	6.95	7.18	7.25	6.60	7.21	7.29	0.16	0.1171
FBW (g)	64.48^d^	69.46^c^	69.82^c^	74.35^b^	76.63^b^	81.04^a^	0.93	< 0.001
CBWG (g)	57.54^d^	62.28^c^	62.57^c^	67.75^b^	69.42^b^	73.75^a^	0.94	< 0.001
ADG (g)	0.59^d^	0.64^c^	0.64^c^	0.69^b^	0.71^b^	0.75^a^	0.01	< 0.001
SGR (% d^−1^)	2.28^c^	2.33^bc^	2.30^c^	2.41^ab^	2.47^a^	2.48^a^	0.03	< 0.001
RGR (g/g)	8.47^b^	8.87^b^	8.81^b^	9.82^a^	10.33^a^	10.53^a^	0.28	< 0.001
FCR (g/g)	1.42	1.39	1.66	1.28	1.38	1.21	0.14	0.2777
PER (g/g)	2.35^c^	2.41^c^	2.41^c^	2.62^b^	2.62^b^	2.77^a^	0.04	< 0.001

IBW, initial body weight; FBW, final body weight; CBWG, cumulative body weight gain; ADG, average daily gain; SGR. Specific growth rate; RGR, relative growth rate; FCR, feed conversion ratio; PER, protein efficiency ratio.

One-way ANOVA non-significant for P > 0.05. The different letters within the same raw indicated to significance variation. Data were presented as the mean ± pooled standard error.

### Fish body chemical analysis

Dietary supplementation significantly altered the moisture content, dry matter, crude protein, crude lipid, and crude ash of the total body of fish among the treated groups (*P* > 0.001) (Table 4). When dietary supplements BG were paired with a high amount of BC, the moisture content, crude fat, and crude ash were significantly reduced compared to the control group. Conversely, fish fed high doses of *B. coagulans* alone or in combination with BG had considerably higher crude protein and dry matter levels in their bodies.

**Table 4:** Effects of dietary supplementation with β-glucan and/or *Bacillus coagulans* on whole body composition (% wet weight basis) of tilapia fish.

**Parameters**	**Dietary treatments**						**Pooled *SEM***	***p*-value**
	**CNT**	**BG**	**BC1**	**BC2**	**BG+BC1**	**BG+BC2**		
Moisture	73.64^a^	73.83^a^	73.08^b^	72.57^bc^	72.58^bc^	72.41^d^	0.16	< 0.001
Dry matter	26.36^c^	26.17^c^	26.92^b^	27.42^ab^	27.42^ab^	27.59^a^	0.14	< 0.001
CP	11.86^e^	12.86^cd^	14.01^cb^	15.27^a^	14.59^ab^	15.42^a^	0.12	< 0.001
CF	7.87^a^	7.51^ab^	7.16^bc^	7.05^c^	7.16^bc^	6.87^c^	0.13	0.002
Ash	5.62^a^	5.21^b^	4.66^c^	4.48^cd^	4.45^cd^	4.28^d^	0.08	< 0.001

CP, crude protein; CF, crude fat.

One-way ANOVA non-significant for P > 0.05. The different letters within the same raw indicated to significance variation. Data were presented as the mean ± pooled standard error.

### Blood hematological differentiations

Red blood cell, hemoglobin, and PCV concentrations were significantly (*P* < 0.05 or 0.001) improved in all fish groups fed any dose of BC alone or in combination with BG compared to other treatments (Table 5). Moreover, the highest RBC, Hb, and PCV levels were found in the tilapia group fed BG with a low dose of BC. In the same context, all fish groups fed BC1 alone or in combination with BG significantly (*P* ? 0.05 or 0.001) increased white blood cell counts, a lymphocyte level, and lymphocyte percentage compared to other treatments. While, dietary supplements did not affect MCV, MCH, MCHC, monocytes, eosinophil, or basophil levels.

**Table 5:** Effects of dietary supplementation with β-glucan and/or *Bacillus coagulans* on blood hematological measurements of tilapia fish.

**Parameters**	**Dietary treatments**						**Pooled SEM**	***p-*value**
	**CNT**	**BG**	**BC1**	**BC2**	**BG+BC1**	**BG+BC2**		
**Erythrocyte's constituents**
RBCs (× ^10^/mm3)	3.09^c^	3.15^c^	3.28^b^	3.34^b^	3.58^a^	3.31^b^	0.03	0.001
Hb (g/100 ml)	9.42^c^	9.50^c^	9.96^b^	10.20^b^	10.84^a^	10.0^b^	0.07	< 0.001
PCV (%)	30.5^c^	31.0^c^	33.0^b^	33.50^b^	33.50^a^	33.0^b^	0.54	0.005
MCV	94.90	94.78	96.97	96.7	95.86	96.07	0.97	0.557
MCH	30.30	30.04	30.20	30.36	30.16	30.05	0.19	0.764
MCHC	31.66	31.43	30.89	31.15	31.19	31.01	0.39	0.749
**Leucocyte's constituents**
WBCs (× 103/mm3)	10.3^d^	10.5^cd^	12^abc^	12.5^ab^	13.4^a^	11.6^bcd^	0.46	0.019
Het%	18.87^a^	13.33^bc^	10^de^	12.8^cd^	8.96^e^	15.52^ab^	0.79	0.001
Lym%	68.93^c^	74.29^b^	78^a^	75.14^ab^	78.36^a^	73.28^bc^	1.00	0.005
Mon%	8.4	8.95	9	8.56	9.7	7.5	0.79	0.455
Esin%	1.9	1.5	1.5	1.5	1.49	1.98	0.41	0.489
Bas%	1.9	1.93	1.5	2	1.49	1.72	0.41	0.489
Het (× 103/mm3)	1.93^a^	1.4^b^	1.2^b^	1.6^ab^	1.2^b^	1.8^a^	0.12	0.019
Lym (× 103/mm3)	7.1^d^	7.8^cd^	9.36^ab^	9.39^ab^	10.5^a^	8.5^bc^	0.32	0.003
Mon (× 103/mm3)	0.87	0.94	1.08	1.07	1.3	0.87	0.12	0.171
Esin (× 103/mm3)	0.20	0.16	0.18	0.18	0.20	0.23	0.05	0.848
Bas (× 103/mm3)	0.20	0.20	0.18	0.25	0.20	0.20	0.05	0.568

RBCs, red blood cell count; Hb, hemoglobin; PCV, packed cell volume; MCV, mean corpuscular volume; MCH, mean corpuscular hemoglobin; MCHC, mean corpuscular hemoglobin concentration; WBCs, white blood cell count; Het, Heterophil; Lym, Lymphocyte; Mon, Monocyte; Esin, Eosinophil; Bas, Basophil.

One-way ANOVA non-significant for *P* > 0.05. The different letters within the same raw indicated to significance variation. Data were presented as the mean ± pooled standard error.

### Blood biochemical profile, antioxidant and immunity parameters

Results of serum biochemical profiles, oxidative remarks activities and immune response were exhibited in Table 6. The serum total protein, albumin, and globulin levels in the BG plus BC1 group were significantly higher than in the other treated groups (*P* > 0.05 or 0.001). At the same time, the control and all supplemented groups had no significant (*P* > 0.05) difference in AST, ALT, total glyceride, cholesterol, and glucose levels.

**Table 6:** Effects of dietary supplementation with β-glucan and/or *Bacillus coagulans* on blood biochemical profile, oxidative remarks and immune activity of tilapia fish.

**Parameters**	**Dietary treatments**						**Pooled SEM**	***p-*value**
	**CNT**	**BG**	**BC1**	**BC2**	**BG+BC1**	**BG+BC2**		
**Biochemical profile**
TP (g/dl)	3.90^cd^	3.94^bc^	3.92^c^	3.81^d^	4.35^a^	4.03^b^	0.03	< 0.001
ALB (g/dl)	2.02^b^	2.02^b^	1.98^bc^	1.86^c^	2.19^a^	2.06^ab^	0.04	0.022
GLOB (g/dl)	1.88^b^	1.92^b^	1.94^b^	1.95^b^	2.16^a^	1.97^b^	0.03	0.004
ALT (U/l)	38.47	36.28	35.23	35.81	33.72	36.11	1.15	0.246
AST (U/l)	29.89	28.94	29.71	29.00	28.86	29.63	0.58	0.701
TGly (mg/dl)	91.37	93.03	93.17	94.5	92.39	93.18	1.65	0.839
ChoL (mg/dl)	79.48	81.55	81.27	82.03	80.25	83.10	1.85	0.779
Glu (mg/dl)	63.32	63.64	63.67	63.11	62.54	62.77	0.59	0.693
**Immune parameters**
Phag. activity%	9.21^d^	9.46^d^	9.96^c^	10.92^a^	10.44^b^	9.49^d^	0.11	< 0.001
Phag. index	1.09	1.12	1.15	1.26	1.09	1.06	0.06	0.336
LYZ (μg/ml)	8.3^c^	8.90^c^	9.74^bc^	10.60^ab^	11.52^a^	9.91^abc^	0.46	0.022
IgM (μg/ml)	4.14	5.10	5.3	4.97	5.7	5.07	0.31	0.128
**Oxidative remarks**
Cortisol (ng/ml)	31.49	33.68	33.91	34.11	35.38	32.27	3.76	0.977
SOD (IU/l)	10.66	11.90	12.89	11.68	13.37	12.38	0.71	0.240
CAT (IU/l)	13.03^b^	14.24^b^	14.44^ab^	14.36^ab^	15.76^a^	13.35^b^	0.40	0.029
MDA (IU/l)	17.03^a^	14.66^bc^	13.58^c^	10.94^d^	11.50^d^	16.22^ab^	0.59	0.002

TP, total protein; ALB, albumin; GLOB, globulin; ALT, alanine amino transferase; AST, aspartate amino transferase; TGly, total glysride; ChoL, cholesterol; Glu, glucose; Phag, phagocytes; LYZ, lysozyme; IgM, immunoglobulin M; SOD, super oxide dismutase; CAT, catalase; MDA, malonaldehyde.

One-way ANOVA non-significant for *P* > 0.05. The different letters within the same raw indicated to significance variation. Data were presented as the mean ± pooled standard error.

According to the immunity data presented in Table 6, the serum phagocytic activity was significantly (*P* > 0.001) stimulated in the BC2 group, followed by the BG+BC1 fish group, in which the effects of BC supplementation alone or in combination with BG significantly (*P* > 0.05) activated lysozyme concentrations compared to the other two groups. Whereas no significant variances in phagocytic index or IgM concentrations were detected between the treatment and control groups (*P* > 0.05).

According to the oxidative remarks findings in Table 6, the BC2 and BG+BC1 groups had the lowest MDA content. In contrast, the CAT level in the BG+BC1 group was significantly (*P* > 0.05) higher than in the other groups. While, cortisol and SOD activity levels had no significant effects on any of the groups studied.

### Digestive enzymes and intestinal histomorphometry indices

The digestive enzymes and morphometric indices of the intestine were influenced by supplementation with β-glucan and/or *B. coagulans* (Table 7). There are no significant (*P* > 0.05) variations in amylase and lipase activity across all treated and non-treated groups.

**Table 7:** Effects of dietary supplementation with β-glucan and/or *Bacillus coagulans* on intestinal digestive enzymes and histomorphometry features of tilapia fish.

**Parameters**	**Control Dietary treatments**						**Pooled SEM**	***p-*value**
	**CNT**	**BG**	**BC1**	**BC2**	**BG+BC1**	**BG+BC2**		
**Digestive enzymes**
Amylase (U/L)	103.7	100.5	103.9	104.1	107.5	106.7	3.77	0.807
Lipase (U/L)	82.9	85.49	86.61	93.83	91.17	82.24	3.05	0.173
**Histomorphometrically characterization**
VL (μm)	148.5^f^	158.4^e^	192.3^d^	226.9^b^	274.1^a^	201.7^c^	2.19	< 0.001
CD (μm)	25.76^a^	24.53^ab^	23.16^ab^	22.03^b^	21.10^b^	21.3^b^	1.02	0.040
VL/CD	5.32^d^	6.64^c^	9.60^b^	9.84^b^	11.04^a^	10.61^a^	0.15	< 0.001
VW (μm)	58.55^b^	59.41^b^	64.90^a^	64.26^a^	65.52^a^	59.06^b^	1.27	0.004
IVS (μm)	22.42^a^	19.71^b^	17.47^c^	16.82^cd^	15.25^e^	16.09^de^	0.31	< 0.001
VSA (μm^2^)	9123.0^c^	9602.6^bc^	7612.6^c^	12179^abc^	14723^a^	13993^ab^	3.95	0.020
GC count/mm^2^	18.9^f^	19.96^e^	24.23^d^	27.33^c^	31.23^a^	30.26^b^	0.30	< 0.001

VL, Villi length; CD, Crypt depth; VW, Villi width; IVS, Inter-villi space; VSA, Villi surface area; GC, Goblet cells.

One-way ANOVA non-significant for *P* > 0.05. The different letters within the same raw indicated to significance variation. Data were presented as the mean ± pooled standard error.

Owing to the histomorphometrically features of small intestine, the villus length increased significantly (*P* > 0.001) when BG was combined with BC1. However, the crypt depth and inter villi space decreased significantly (*P* > 0.05) in all fish supplemented groups. The fish-fed supplemented diets with BG and any quantity of BC had the lowest crypt depth and inter villi spacing values. In contrast, the villus length per crypt depth ratio improved significantly (*P* > 0.001) in fish groups fed BG supplemented diets in combination with any amount of BC compared to the control group. Conversely, villus width increased significantly (*P* > 0.01) in fish groups receiving BC supplemented diets alone or in combination with BG compared to the control group. Compared to other groups, the villus surface area and goblet cell count significantly (*P* > 0.01 or 0.001) improved in fish fed BG+BC1, BG+BC2, and BG2, respectively.

### Anterior intestinal morphometric structures

**Figure 1:**
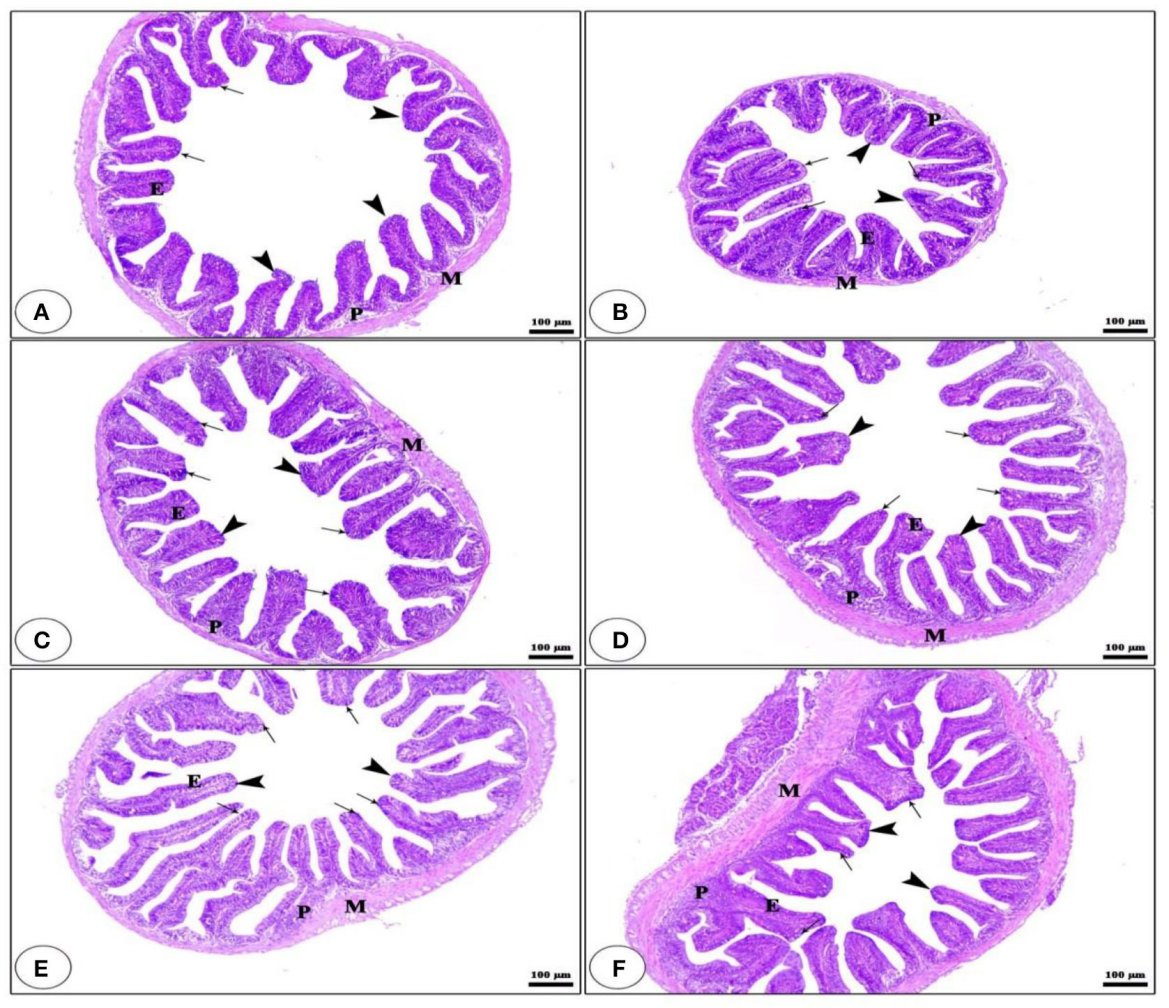
Histological description for transversal section photomicrograph of *O. niloticus* anterior part of intestine. While, CNT **(A)**, BG **(B)**, BC1 **(C)**, BC2 **(D)**, BG + BC1 **(E)**, and BG + BC2 **(F)**. The intestinal villi (arrow heads) with intact simple columnar epithelium (E) rested on lamina propria of loose connective tissue (p) and lamina muscularis (M) with numerous goblet cells (arrows) present in the lamina epithelia. The examined sections stained with H&E (× 100 μm).

Light microscopy has been applied to estimate the morphological features of the anterior section of the small intestine (Figure 1). The histological examination of the intestinal villi revealed a higher length and normal structure, which confirms that the tilapia intestine is out of inflammation. The fish group that received BG + BC1 supplemented diets had the maximum villus length compared to other groups.

### m-RNA gene expression

**Figure 2:**
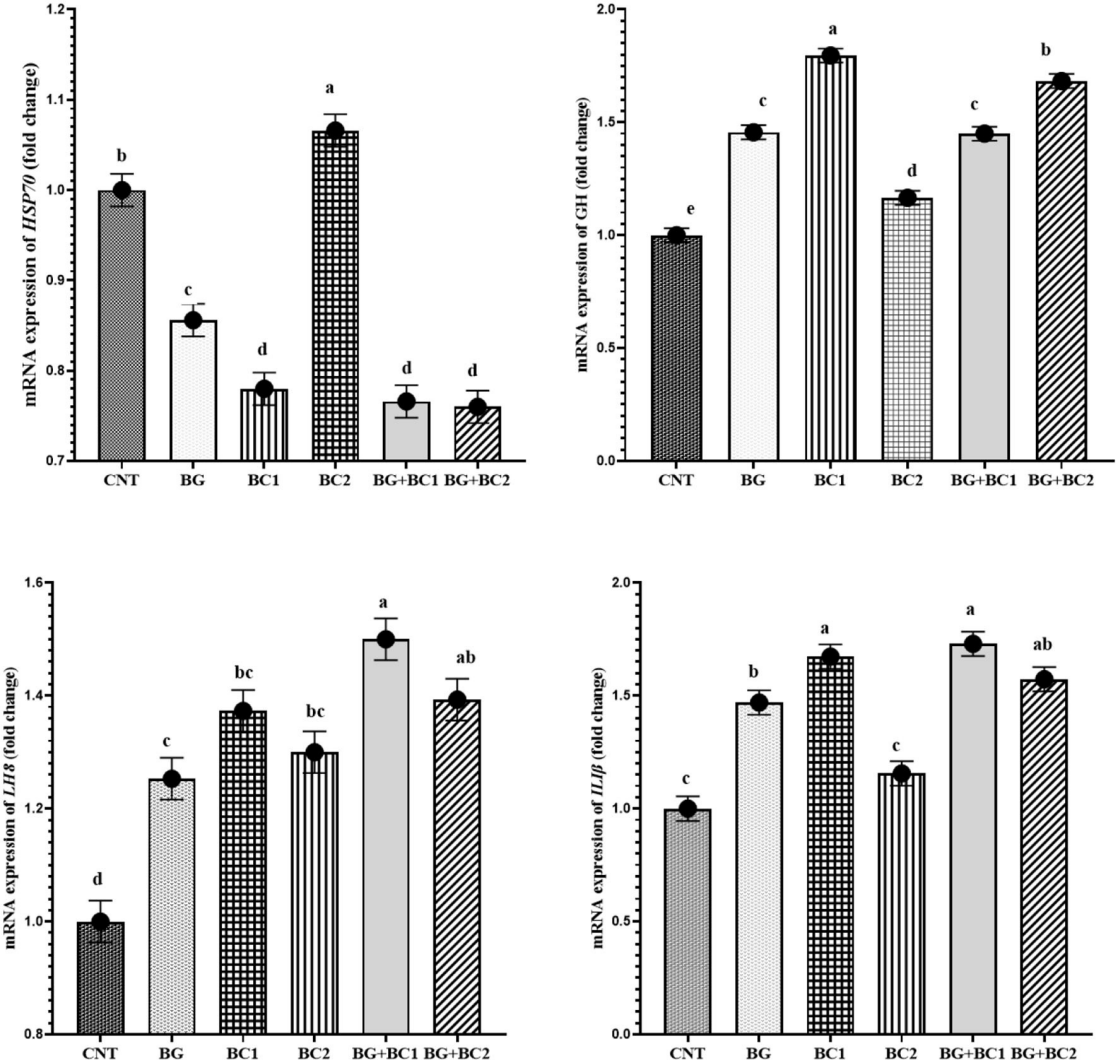
*mRNA* expression levels of heat shock protein*-70* (*HSP70*), growth hormone (*GH*), interleukin-1 beta (*IL-I*β) and interleukin-8 (*IL-8*) genes in the liver tissue of Nile tilapia, *Oreochromis niloticus* (mean ± MSE) fed on diets supplemented with graded levels of β-glucan and/or *Bacillus coagulans* for 14 weeks. Columns with different superscribt letters are significantly different (*P*<*0.05*).

The mRNA level of the HSP7 gene was significantly lowered in all supplemented diets, with the maximum significance decrease observed under the impact of BG+BC1 or BC2 dietary supplementation compared to the control group (Figure 2). In contrast, feeding BG or/and BC elevated *GH, IL-1*β, and *IL-8* transcription compared to the control group. However, high levels of *GH* transcription were identified in fish fed low levels of BC, whereas *IL-1*β levels increased under the impacts of BC1 alone or in combination with BG compared to the control group and other treated groups. Finally, *IL-8* transcription was activated at a higher level in fish groups that received BG in combination with any amount of BC compared to other experimental groups.

## Discussion

Although numerous research has been conducted on applying β-glucan or probiotics in tilapia diets (59), the current trial is the first to describe the relevance of employing both β-glucan and *B. coagulans* on tilapia growth and performance. The feeding trial findings showed that incorporating BG and *B. coagulans* in *O. niloticus* diets significantly influenced growth performance indices. The BG/BC mixture contains β-glucan and *B. coagulans*, associated with increased growth and feed efficiency in fish. While, the current experiment merge between BG and BC in promoting *O. niloticus* growth is owing to BC's function in boosting the variety of beneficial bacteria in the gastrointestinal tract of fish (60). The development of beneficial bacterial strains in the digestive tract facilitates nutrient digestion and absorption through epithelial cells (61).

Furthermore, dominating the beneficial microorganisms in the intestinal microbiota reduces the influence of pathogenic bacteria on intestinal immunity (62). Thus, the general body's immune system is connected to the intestine's immunity and health (63). The same effects are generated by prebiotics, specifically β-glucan, which encourage the development and activity of the desirable healthy intestine microbial population while suppressing pathogen colonization and preventing inflammation (64). Based on this hypothesis, both BG and BC synergistically impacted the performance of *O. niloticus*.

The possible significance of BG/BC may also be linked to their function in enhancing feed intake by promoting feed palatability, which subsequently leads to enhanced feed efficiency (20, 32). Feeding *O. niloticus* with *Bacillus* sp. improved growth performance and feed efficiency (65), whereas feeding with BG also affected growth rates, as previously documented by Pilarski et al. (20).

The improved feed efficiency may be explained by referring to how BG/BC affects gastrointestinal morphometry indices (66). The findings showed that feeding fish BG and BC positively impacted the length, surface area and width of the intestinal villi and the number of goblet cells. These findings support the synergistic role of both BG and BC in increasing the absorption capacity of intestinal barriers, allowing enough quantities of nutrients to be digested for biological and metabolic activities in fish tissues (1, 67). Furthermore, the increased number of goblet cells is linked to their function in defending the intestinal membranes from dangerous germs by producing glycoprotein and antibacterial substances (68). Likewise, Ghalwash et al. (69) study demonstrated that *Bacillus* spp. dietary incorporation into tilapia diets improved all intestinal histomorphometric parameters.

Feed additives are often claimed to be the main cause of enhanced or reduced hematological and biochemical variables in fish compared to normal values (70). The findings showed that fish fed BG and BC, alone or in combined form had normal biochemical levels that were regarded within the typical range for healthy fish. In parallel, fish fed BG/BC1 had increased total protein, albumin, and globulin levels compared to other treated groups. Moreover, it has been observed that a low level of *Bacillus* spp. in combined with beta-glucan increases the total protein contents in Nile tilapia (71, 72). Thus, supplementing fish feed with probiotics or prebiotics promotes intestinal immunological responses against pathogens, including humoral and cell-mediated responses, results in an increment in immunoglobulin levels in the blood and an increase in total protein (73).

Phagocytes are white blood cells continuously generated from the bone marrow and have been identified as responsible for removing dead cells and invading bacteria (74). Furthermore, white blood cells are part of the cellular-immune system, which protects the fish body from infectious diseases (75). Moreover, lysozyme, a small cationic protein, destroys or kills bacteria by lysing their cell wall peptidoglycan, breaking bacterial membranes, and activating autolytic enzymes in the bacterial cell wall (76). Our research findings concluded that the impact of BC alone or combined with BG supplemented diets boost cellular-immune activity. Major probiotic mechanisms of action involve epithelial barrier improvements, enhanced adherence to the intestinal mucosa, and associated inhibition of pathogen adhesion, competitive exclusion of pathogenic bacteria, generation of anti-microorganism molecules, and innate immune system regulation (77). Specifically, probiotic-based diets could stimulate cellular immune response features through the activation of macrophages, natural killer (NK) cells, antigen-specific cytotoxic T-lymphocytes, and the release of various cytokines in a strain-specific and dose-dependent manner (78).

Probiotics and prebiotics are well known for their protective role against oxidative stress in fish (79, 80). Under stressful situations, reactive oxygen species (ROS) may be produced in large quantities, damaging the cellular membrane by causing lipid peroxidation (81). Biologically, the cell triggered many internal anti-oxidative reactions to deal with the harmful effects of ROS on the cellular membrane by boosting the release of anti-oxidative enzymes such as Catalase (CAT), and alleviating the MDA activation (82). The present investigation shows higher CAT activity and lower MDA levels in fish fed BG and BC, consistent with previous reports (1, 67, 83). Kim et al. (84) reported higher CAT activities after being pre-treated with β-glucan for 15 days in grass carp. The increased CAT activities and lower MDA levels might be attributed to distinct activation of up-regulating antioxidant-related enzyme gene expression *via* the antioxidant properties of β-glucan (84). Furthermore, fish administered BC had higher CAT, which the beneficial bacterial population may explain in boosting the overall immune response (85).

When studying the influence of functional feed additives on aquatic organisms, the transcription of some growth, immunological, and stress-associated genes is often used to understand the mechanism of action on a genetic basis (86). Under stress, fish cells secrete high heat shock protein 70 (*HSP70*), which increases protein integrity and reduces apoptosis (87). The current results exhibited *HSP70* downregulated in tilapia fish received diets supplemented with BG/BC combination, which is connected with the possible function of BG and BC in sustaining fish health. Furthermore, the lower mRNA level of the *HSP70* gene indicates that the tilapia is not subjected to stressful rearing conditions (88). Moreover, it is well known that growth hormone (GH) regulates various key physiological activities in fish, such as mineral homeostasis, growth, and metabolism (89). The findings of our study showed that the impacts of BC alone or in combination with BG resulted in increased *GH* expression levels in tilapia fish. These findings were consistent with other previous reports (20, 90). Probiotics significantly altered the expression of growth-related genes, demonstrating a desirable influence of these probiotics in overall fish metabolic activities (4, 91). Interleukin genes were estimated to maintain growth, differentiation, and activation during inflammatory and immunological responses (92). The gene findings showed that fish fed BG/BC had higher levels of interleukin-1 beta (*IL-I*β) and interleukin-8 (*IL-8*). The activation of the *IL-8* and *IL-I*β genes in response to BG and BC supplemented diets verified the synergistic protective potential function of these mixtures in attracting and activating neutrophils in inflammatory regions and promoting the immune system response function and overall health status of fish (17, 93).

## Conclusion

Finally, the current findings revealed that dietary supplementation with *B. coagulans* alone or combined with beta glucan might improve tilapia growth performance. Furthermore, supplementation of feed additives (β-glucan and *B. coagulans*) might boost fish health by promoting immune responses and antioxidant capacity and altering some associated blood biochemical and hematological parameters in tilapia.

## Data availability statement

The all data presented in the study are available under reasonable request from the corresponding author.

## Ethics statement

The animal study was reviewed and approved by the Ethical Principles of the Experimental Animal Welfare Ethics Committee of Zagazig University (No. ZU-IACUC/2/F/110/2022).

## Author contributions

AF, IE-R, AN-A, AE-R, and AE designed the experiment and drafted the manuscript. AE-R, MA-E, and MN conducted the trial, sample collection, analysis, data collection, and wrote the manuscript. KM, HS, MS, MJ, and MA assisted in sample collection, induced breeding, data analysis, and revised the final drafted manuscript. All named authors have significantly contributed toward the final version of this research study and approval for publication.

## Conflict of interest

The authors declare that the research was conducted in the absence of any commercial or financial relationships that could be construed as a potential conflict of interest.

## Publisher's note

All claims expressed in this article are solely those of the authors and do not necessarily represent those of their affiliated organizations, or those of the publisher, the editors and the reviewers. Any product that may be evaluated in this article, or claim that may be made by its manufacturer, is not guaranteed or endorsed by the publisher.
